# A combination containing natural extracts of clove, Sophora flower bud, and yam improves fertility in aged female mice *via* multiple mechanisms

**DOI:** 10.3389/fendo.2022.945690

**Published:** 2022-11-22

**Authors:** Wei Liu, Xinyu Wang, Yating Chen, Huiyu Zhang, Jing Chen, Jing Zhang, Tinghe Wu, Jing Li

**Affiliations:** ^1^ State Key Laboratory of Reproductive Medicine, Nanjing Medical University, Nanjing, Jiangsu, China; ^2^ Reproductive Research and Development Center, Hainan Leyun Biotechnology Co., Ltd., Qionghai, Hainan, China; ^3^ Tinghe Wu, State Key Laboratory of Translational Medicine and Innovative Drug Development, Jiangsu Simcere Pharmaceutical Co., Ltd., Nanjing, Jiangsu, China; ^4^ Department of Biotechnology and Biomedicine, Yangtze Delta Region Institutes of Tsinghua University, Jiaxing, Zhejiang, China

**Keywords:** healthy product, antioxidant factors, ovary, fertility, aging

## Abstract

**Introduction:**

With society development, the age at which women choose to have children has been gradually delayed. To improve the reduced fertility in women at advanced maternal age, we developed a combination containing natural extracts from clove, Sophora flower bud and Chinese yam with a mass ratio 15:6:10 and named it as DACHAO.

**Methods and Results:**

We then gavage DACHAO at a dose of 310 mg/kg BW to female mice at 10 month of age and investigated its effects on ovarian functions. Using MitoTracker probes, ROS, and JC-1 staining, we found that DACHAO treatment improved mitochondria functions in oocytes from aged mice. We also observed increased blastocyst formation when mature oocytes from control and DACHAO treated mice were for IVF and in vitro embryo culture. Cell counting and TUNEL assay further revealed increased cell numbers and decreased apoptosis in blastocysts of DACHAO group. After control or DACHAO treated mice being mated with fertile male mice, fertility test revealed a greater first litter size in the DACHAO group. Further studies demonstrated that DACHAO treatment could alleviate the retarded ovarian function in aged mice via changes in serum hormone levels, over-expression of antioxidant factors, under-expression of inflammation-related factors, and reduced apoptosis in the ovaries.

**Discussion:**

Thus, the new combination DACHAO will be a good choice in clinic to improve ovarian functions for women at advanced maternal age.

## Introduction

Due to the growing numbers of late marriages and childbearing, especially following the two-child and three-child policies in China, the proportion of pregnancies at advanced age has further increased. Older women with reduced fertility are willing to seek help by assisted reproductive technology (ART) (1). However, the success rate of assisted reproductive technology decreases significantly with age advancement. A recent multi-center retrospective study in Chinese population indicated a downside effect on live birth rate (LBR) after *in vitro* fertilization and intracytoplasmic sperm injection treatment from 59.74% at the age under 30 years to only 24.36% for the age over 37 years (2). Thus, assisted reproductive technology is not the master key to solve the reproductive problems of aged women. The attention of researchers has been focused on the discovery of approaches to help women at advanced maternal age to improve their reduced fertility.

The cessation of menstruation is the result of ovarian aging. Due to an age-related follicular reserve decrease, the menstrual cycle becomes irregular, eventually leading to the Menopause (3). Human ovaries have a number of primordial follicles that is fixed at birth, which steadily declines throughout life due to atresia and continuous recruitment towards ovulation (4). The peak number of 6 to 7 million follicles is reached at 20 weeks of gestation. Then, the number of oocytes decreases sharply, resulting in only 300,000 to 400,000 remaining at birth (5, 6). During the reproductive lifespan, the continued growth of primordial and primary follicles into secondary and larger follicles results in a gradual reduction in the original follicle pool. In addition, the primordial follicle pool may also shrink due to the apoptosis of the resting follicles (7, 8). According to the current knowledge about the human female circadian clock, a woman’s fertility begins to decline in the early 30s, with a steep decrease beginning after the age of 35 (3, 9). The decreased fecundity during ovarian aging accompanies with the declines in both oocyte quantity and quality. Multiple mechanisms have been proposed to be the explanation which includes the free radical theory, apoptosis, telomere shortening, mitochondrial dysfunction or inflammation (10–14). Among these theories, the studies conducted on the free radical theory are particularly extensive. As mentioned below, the aging process is characterized by high concentrations of endogenous reactive oxygen species but the antioxidant defense activity is limited which leads to oxidative injuries, such as lipid peroxidation of the cell membranes, enzyme inactivation, protein oxidation, and DNA damage (15–17). Oxidative stress in the aged ovary has been reported to be related with follicular atresia, oocyte meiotic defects, shortened telomeres, or detrimental oocyte fertilization and embryonic development (18, 19). Meanwhile, the anti-aging effects of antioxidants, such as melatonin, coenzyme Q10 and glutathione have also been reported in improving female reproduction at advance maternal age (20–22).

Since ancient times, people have used medical plants for the treatment of various diseases. In modern society, active extracts from herbs are widely used worldwide as complementary and alternative medicines, especially by women, middle-aged or elderly people and individuals with higher levels of education and higher incomes. Many active ingredients from traditional medical plants have now been applied in women with reproductive disorders. For example, soybean isoflavone or diosgenin from Chinese Yam, due to their structural similarity to the precursor of steroid hormones, have been widely used to alleviate menopausal syndrome (23). Hydroethanolic extract of clove bud (*Syzygium aromaticum*) or resveratrol (mainly from grape seeds), both have been shown to increase blastocyst formation in cryo-preserved oocytes (24, 25). Additionally, resveratrol or quercetin, a plant-derived flavonoid from many edible fruits and vegetables were also reported as natural antioxidants in improving follicle development and oocyte quality of aged mice (26). To be noted, most of the aforementioned studies have been focused on the effects of single molecules with high purity and high concentration on the improvement of female fertility. Few reports explored the combinations of different components in the recovery of ovarian function. As we know, flos sophora, clove bud and Chinese yam are traditional Chinese medicinal and edible plants for their good taste and nutritional value (27–29). Based their potential regulations on ovarian functions, in the study, we aimed to develop a healthy product which contained active extracts from three kinds of Chinese herbs, Flos sophora (quercetin, 95%), clove bud (Hydroethanolic extract, 90%) and Chinese yam (diosgenin, 15%), and mixed at the ratio (15:6:10) to examine its effects on the ovarian function and fertility in aged mice.

## Materials and methods

### Preparation of DACHAO

**Figure 1:**
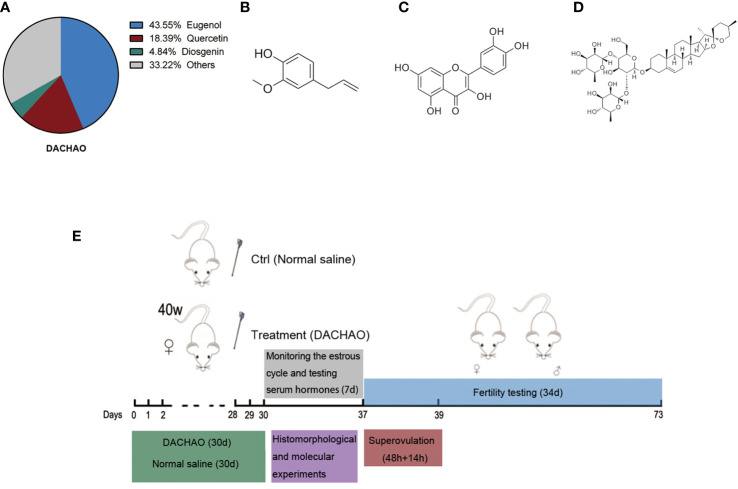
Establishment of a gavage model in aged female mice and DACHAO characterization. **(A)** Aging mice models were established in the experiment: female mice at 40 weeks of age were administrated with DACHAO (310 mg×kg- 1) daily for a month. In control group, mice were gavaged with the same volume of normal saline (green region). After gavage, the mice were tested for estrous cycle immediately. Blood samples were collected from mice during the diestrus randomly to detect serum hormone levels (gray region). The remaining mice were randomly divided into 3 parts. A random selection of 5 mice were co-caged mating for fertility testing (blue region). A random selection of 8 mice were euthanized and the ovaries of them were collected for histomorphological and molecular experiments (purple region). The remaining mice were superovulated and euthanized in batches when needed, then oocytes were collected for experiments (red region). **(B)** *Via* high performance liquid chromatography test and calculation, the proportion of each main component in DACHAO. **(C–E)** Chemical structures of eugenol, quercetin, and diosgenin.

We commissioned the company (Si Wei Tech Bio Co., Ltd, Zhengzhou, Henan, China) to mix clove extract, Sophora flower bud extract and Chinese yam extract in a mass ratio of 15:6:10 to make DACHAO. The content of eugenol in clove extract was 90%, the content of quercetin in Sophora flower bud extract was 95%, and the content of diosgenin in Chinese yam extract was 15%. All the active contents were qualified by high performance liquid chromatography (**Figure 1A**). The chemical structural formulas of these bioactive compounds are displayed in **Figures 1B–D**. Thus, in every 100 mg of DACHAO, the content of eugenol is 43.55 mg, the content of quercetin is 18.39 mg, and the content of diosgenin is 4.84 mg. The ratio of this product is determined according to gavage dosage of each component per mouse. The dosage of each plant extract on mouse refers to the previous reports (30–33).

### Experimental animals

All mice were obtained from the Model Animal Research Center of Nanjing Medical University (Nanjing, Jiangsu, China) and housed in the animal facility at Nanjing Medical University. Mice were maintained on a 12-h light: 12-h darkness cycle and were provided with food and water ad libitum. Except for mice in mating trails, others were group housed with up to five mice per cage. Adult male B6D2F1 mice (10–14 weeks old) were chosen as sperm donors for IVF experiment. All experiments requiring the use of animals were approved by the Committee on the Ethics of Animal Experiments of Nanjing Medical University.

### Animal experiments and diets

40W ICR female mice were randomly divided into control group and treatment group, with 60 mice in each group. In addition to the daily diet, mice were gavaged one time at 10:00 am every day. In treated group, each mouse was gavaged with 0.5 ml normal saline contained DACHAO (the plant extracts) at a dose of 310 mg/kg BW. In control group, mice were gavaged with the same volume of normal saline. All mice were treated for one month. At the end of the experiment, serum from 8 mice in the interphase were collected for relevant testing. A random selection of 5 mice were co-caged mating for fertility testing. A random selection of 8 mice were euthanized and the ovaries of them were collected for histomorphological and molecular experiments. The remaining mice were superovulated and euthanized in batches when needed, then oocytes were collected for experiments (**Figure 1E**). To test the biosafety of DACHAO, we additionally prepared ten 40W aged female ICR mice to gavage with 2 times the dose of DACHAO for one month. After the gavage, serum from 5 mice in the interphase were collected and detected the liver and kidney function and blood lipid-related indicators with the DACHAO group and the control group.

### Superovulation

48 hours after intraperitoneal injection with eCG, the mice were intraperitoneal injected with hCG. 14 hours after the hCG injection, the mice were euthanized and the ovulated oocytes were collected in the ampulla of the fallopian tubes from both sides.

### Immunoblotting analysis

Ovarian proteins were extracted by radioimmunoprecipitation assay (RIPA) lysis buffer (P0013B, Beyotime Institute of Biotechnology) containing protease inhibitor cocktails (M221, Amresco). Protein was quantified by using the BCA protein quantification kit (BI-WB005-500T, Biochannel, Nanjing, China). A total of 10 ug of proteins in each sample was loaded and separated by electrophoresis (165-8000, Bio-Rad, USA). Proteins were separated by electrophoresis and then electronically transferred to polyvinylidene fluoride membranes. The membranes were blocked in 5% skimmed milk-TBST (TBS containing 0.1% Tween 20) for 60 min and incubated overnight at 4°C with specific antibodies. After washing with Tris-buffered saline with Tween 20 (TBST) (5 mL) three times, the horseradish peroxidase (HRP)-conjugated relative secondary antibodies were then used to detect proteins through enhanced chemiluminescence (RPN2232, GE Healthcare, USA) on the Tanon 5200 analysis system. Antibodies against PARP (9542, CST), cleaved-PARP (5625, CST), caspase-3 (9665, CST), cleaved-caspase-3 (9664, CST), CAT (D122036, Sangon Biotech, Shanghai, China), GSS (D154022, Sangon Biotech, Shanghai, China), SOD-1(Bs6057, Bioworld, Shanghai, China) were all rabbit antibodies. Antibodies against β-Actin (YFMA0052, YIFEIXUE, Nanjing, China) were all mouse antibodies.

### Immunohistochemistry

Mouse ovaries were fixed in 10% buffered formalin for paraffin embedding and sectioning. After deparaffinization and rehydration, sections were processed for blocking of endogenous peroxidase activity and antigen retrieval pretreatment. To detect the expression of PCNA, immunohistochemical analyses were performed using a Histo stain Histostain Kit (856743, Invitrogen) with antibodies against PCNA (13110, CST) overnight at 4C. Diaminobenzidine (DAB) reagent was used for coloration on the second day. Non-immune immunoglobulin G (IgG) was applied as a negative control and antigen retrieval pretreatment.

### ROS and mitochondrial membrane potential measurement and mitochondrial distribution detection

To determine the ROS content, mitochondrial membrane potential and mitochondrial distribution of oocytes, the procedure was conducted according to the protocols with the ROS kit (50101ES01, Yeasen, Shanghai, China), the JC-1 assay kit (40706ES60, Yeasen, Shanghai, China) and the MitoTracker (40740ES50, Yeasen, Shanghai, China), respectively. First, MII oocytes were collected from ampulla of fallopian tube and immediately washed with M2 medium 3 times. Then oocytes were transferred into M2 medium containing staining solution and incubated at 37 C in the dark for 30 min. After that, oocytes were washed with M2 medium to remove the excess staining solution. All of the live oocytes were observed by a laser scanning confocal microscope (LSM800, Zeiss, Germany). The relative fluorescence intensity was calculated by software of ZEN.

### *In vitro* fertilization

For IVF studies, donor sperm was collected from B6D2F1 male mice into human tubal fluid (HTF) media (MR-070-D, Millipore, USA) and incubated under oil for 1 h at 37°C in 5% CO2 for capacitation. MII oocytes were collected from ampulla of fallopian tube and then placed into 200 mL of media with sperm (2–3 ×105/mL) for 8 h. After fertilization, zygotes with clear pronuclei were transferred into fresh HTF media overnight until the two-cell embryonic stage. Two-cell embryos were then cultured in small droplets of KSOM media (MR-020P-5F, Millipore, USA) to blastocyst stage.

### Quantitative real-time PCR

Total RNAs from ovaries were purified by TRIzol reagent (Invitrogen, USA) according to the manufacturer’s protocol. RNA concentrations were measured by a spectrophotometer (NanoDrop 2000c, Thermo Scientific, USA). Subsequently, the FastQuant RT kit (Tiangen Biotech, China) was applied to produce cDNA from RNA. The target gene transcripts (*Amhr, Fshr, Lhr, Star, SOD-1, CAT, GSS, IL-6, TNFα, iNOS*) were detected using SYBR Green mix (Applied Biological Materials, Canada) through an ABI StepOnePlus platform (Thermo Scientific, USA) based on the manufacturer’s protocols. Quantification of various mRNAs was performed by using the actin amplification signal as control. The relative expression of target genes was measured by the 2–ΔΔCT method. All of the primer sequences used are listed in **Table S1**.

### HE staining and follicle counting

Mouse ovaries were collected and fixed in 10% buffered formalin for 12 hours, embedded in paraffin, serially sectioned at a thickness of 5 μm, and were then stained with HE for quantification of follicles at each level. Follicle classification and counting are as follows: Follicles were classified as primordial if they contained an oocyte surrounded by a partial or complete layer of squamous granulosa cells. Primary follicles showed a single layer of cuboidal granulosa cells. Oocytes sur-rounded by more than one layer of cuboidal granulosa cells with novisible antrum were determined to be secondary follicles. Antral follicles possessed a clearly defined antral space and a cumulus granulosa cell layer. Corpora lutea were filled with lutein cells. Follicles were considered atretic if they contained either a degenerating oocyte, disorganized granulosa cells, pyknotic nuclei, shrunken granulosa cells, or apoptotic bodies (34). The results are reported as a percentage of follicles at each level counted per ovary.

### Determination of serum hormone levels in mice

After intraperitoneal injection of anesthesia into mice, about 1 ml of venous blood was withdrawn *via* orbital puncture, and left standing in an EP tube for more than one hour at room temperature. Then the venous blood was centrifuged at 3000 rpm for 10 minutes. The supernatant we collected was serum. The collected serum was divided into two parts. One part was measured by AMH Elisa kit according to the manufacturer’s instructions (YIFEIXUE, YFXEM00802, China). The other part was frozen at -80°C and sent to Beijing Northern Biotechnology Research Institute Co., Ltd to detect serum FSH and E2 hormone levels (35).

### TUNNEL analysis

TUNEL immunofluorescence was done by standard kits according to manufacturer’s instructions(40307ES60, Yeasen, Shanghai, China) and were viewed under a laser scanning confocal microscope (LSM 800 META, Zeiss, Germany). TUNEL positive cells were counted by using Image-pro plus 6.0 at different microscopic fields (n=5) for n=3 sections.

### Statistical analysis

GraphPad Prism 6.0 and SPSS 20.0 were used to perform the T test to evaluate differences between groups. Data are mean ± SEM. Statistical significance was set at a probability (p) value< 0.05.

## Results

### DACHAO administration improves both the quantity and quality of oocytes in aged mice

**Figure 2:**
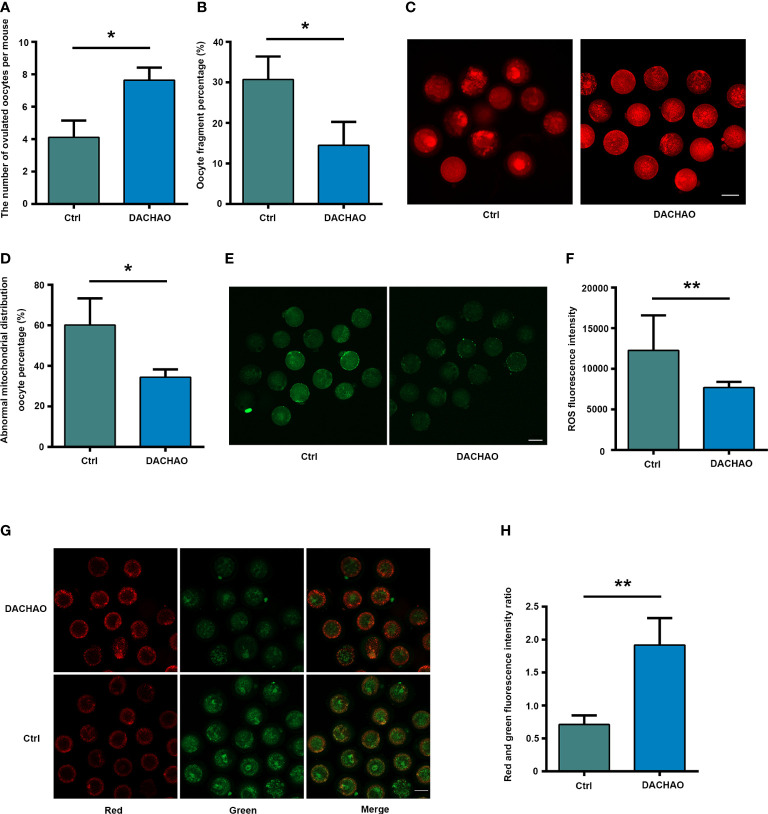
DACHAO administration improves both the quantity and quality of oocytes from aged mice. **(A)** Statistical graph of the number of ovulation after superovulation in each group (n=318). **(B)** Statistical graph of the ratio of oocyte fragmentation after superovulation in each group (n=318). **(C)** MitoTracker staining showed the mitochondrial distribution of oocytes in each group (n=71). **(D)** Statistical graph of abnormal mitochondrial distribution oocyte percentage in each group. **(E)** ROS fluorescence staining showed the level of reactive oxygen species in oocytes in each group(n=60). **(F)** Statistical graph of gray value of ROS fluorescence staining of oocytes in each group. **(G)** JC-1 staining showed mitochondrial membrane potential of oocytes in each group (n=64). **(H)** Statistical graph of the ratio of red-green fluorescence in JC-1 staining in each group. Data are presented as means ± SEM of three independent replicates. *P < 0.05 and **P < 0.01, compared with the control group. n = the total number of oocytes in the 2 groups of 3 repetitions. All bars = 50 μm.

To see the effect of DACHAO compound on the ovarian functions of aged mice. We established a mouse model with the gavage of DACHAO at 310 mg/kg BW to 10-month-old female mice for one month. After finishing the protocol, we found no statistical differences on the body weights between the two groups (**Figure S1A**). The mice in control and treated groups were then given PMSG/HCG for superovulation and mature oocytes were washed from the ampulla of the fallopian tube 14 hours after HCG injection. Ovulated oocyte counting and oocyte fragmentation rate were evaluated and the results showed not only the increased numbers of ovulated oocytes, but also the significant reduction of fragmented oocytes in DACHAO treated mice. In DACHAO treated group, the number of ovulated oocytes per mouse increased from 4.11 to 7.64, meanwhile the fragmentation rate of these oocytes reduced from 30.71 to 14.47 (**Figures 2A, B**). To further assess the ovulated oocyte quality, about oocytes in each group were then labeled with MitoTracker to check the distribution of mitochondria. We found that the ratio of oocytes with abnormal mitochondrial distribution (mitochondria over-concentrated around the nucleus) was reduced in the DAOCHAO-administered group, with the ratio decreasing from 60.19% to 34.40% (**Figures 2C, D**). In another set of experiments, a ROS green fluorescent probe was employed to label and detect the level of oxidative stress in about 30 oocytes of each group. We assessed the ROS green fluorescence intensity and performed statistical analysis. The results showed that DACHAO treatment reduced the senescence-induced increase in the ROS levels in the oocytes (**Figures 2E, F**). A JC-1 fluorescent probe was also used to label and detect the mitochondrial membrane potential in the oocytes, about 30 of each group. We determined the ratio of the red/green fluorescence in two groups of oocytes and DACHAO improved the aging-caused alterations on mitochondrial membrane potential in the oocyte (**Figures 2G, H**).

### The improvement of DACHAO treatment on *in vitro* embryonic development and fertility in aged mice

**Figure 3:**
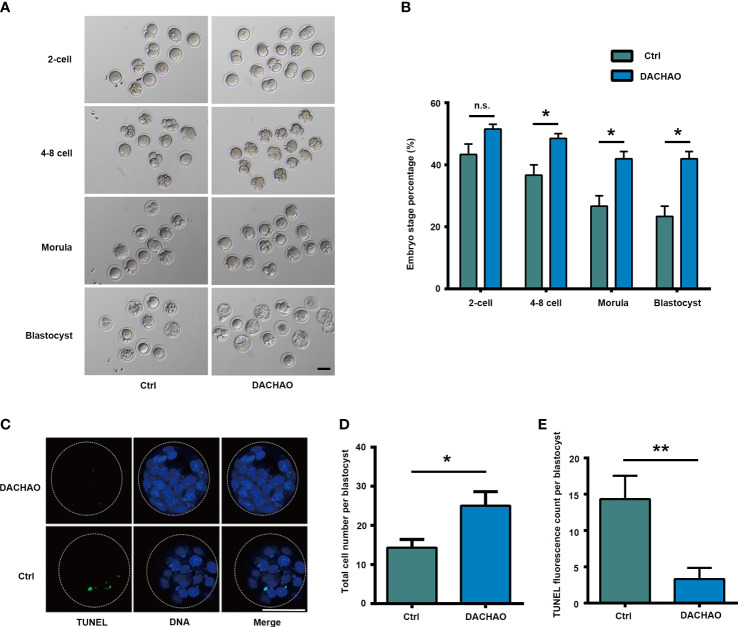
The improvement of DACHAO treatment on *in vitro* embryonic development in aged mice. **(A)** Developmental morphology of embryos at different stages after IVF in each group. **(B)** Statistical graph of the ratios of different stages of embryonic development in each group (n=72). **(C)** TUNEL and Hoechst33342 fluorescence staining of blastocysts in each group. **(D, E)** Statistical graphs of the total number of blastocyst cells and the number of TUNEL fluorescent staining positive signal points in each group (n=16). Data are presented as means ± SEM of three independent replicates. *P < 0.05 and **P < 0.01, and n.s. P≥0.05, compared with the control group. n = the total number of embryos in the 2 groups of 3 repetitions. All bars =50 μm.

Next, IVF and embryo culture were performed to evaluate the developmental potential of oocytes in each group. About 30-50 mature oocytes with good morphology in each group were selected for IVF and the result showed no difference on the rate of 2-cell embryonic development, however, DACHAO treatment showed its beneficial effect on embryonic development which was shown by increased embryonic development at 4–8-cell, morula, and blastocyst stages, as the increasing ratio from 36.67% to 48.48% on the 4-8 cell stage; the increasing ratio from 23.33% to 40.15% on the morula stage; the increasing ratio from 23.33% to 48.48% on the blastocyst stage(**Figures 3A, B**). We then collected blastocysts in each group for TUNEL analysis and Hoechst 33342 were counterstained to label nuclei for cell counting. The results revealed DACHAO treatment significantly decreased apoptosis and increased total cell number in blastocyst. In DACHAO treated group, the number of TUNEL positive cells reduced from 14.33 to 3.33 cells of each blastocyst and the total cell number increased from 14.33 to 25.00 cells of each blastocyst. (**Figures 3C–E**).

**Figure 4:**
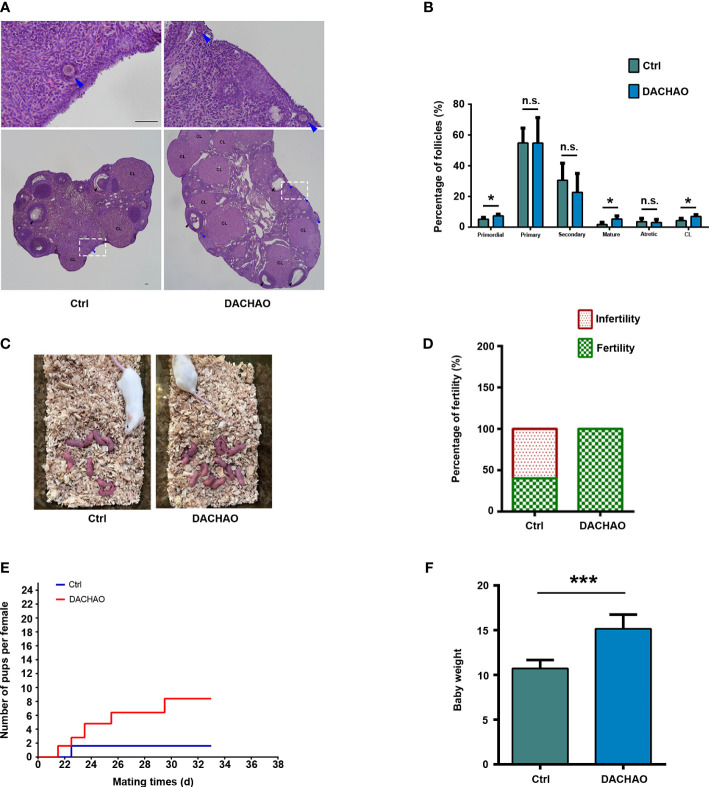
The improvement of DACHAO treatment on fertility in aged mice. **(A)** HE staining of mouse ovarian sections in each group. **(B)** Statistical graph representing the percentage of follicles at different stages in the ovaries from aged female mice in each group. (n=4/group). **(C)** Litters of aged female mice in each group after co-mating. **(D)** Statistical graph of pregnancy percentage in each group of aged female mice (n=5/group). **(E)** Fertility curves of aged female mice in each group (n=5/group). **(F)** Statistical graph of body weight of offspring mice in each group at weaning (n=36). *P < 0.05, ***P < 0.001, and n.s. P≥0.05 compared with the control group. n = the number of mice in each group. All bars =50 μm.

As compared with control group, we observed higher percentages of primordial follicles, growing follicles and corpus luteum of mice ovaries in DACHAO group (primordial follicles: 5.17% *vs*. 7.38%; growing follicles: 1.72% *vs*. 5.28%; corpus luteum: 4.26% *vs*. 6.95%; Ctrl *vs*. DACHAO) (**Figure 4A, B**). To see if the *in vivo* treatment of DACHAO has any effect on fertility of aged mice, the mice in each group were mated with fertile male mice, and their litter size were recorded (**Figures 4C**). As shown in **Figures 4D, E**, in control group, only 40% mice were mated and the average pups was 1.6 per female, whereas all mice treated DACHAO were pregnant with average 8.4 pups delivered. Moreover, the increased weaning weight of the offspring was also observed in DACHAO treated mice (**Figure 4F**). In conclusion, the DACHAO treatment improved the fertility of aged mice.

### DACHAO improves fertility by promoting granulosa cell proliferation and reducing apoptosis in ovaries

**Figure 5:**
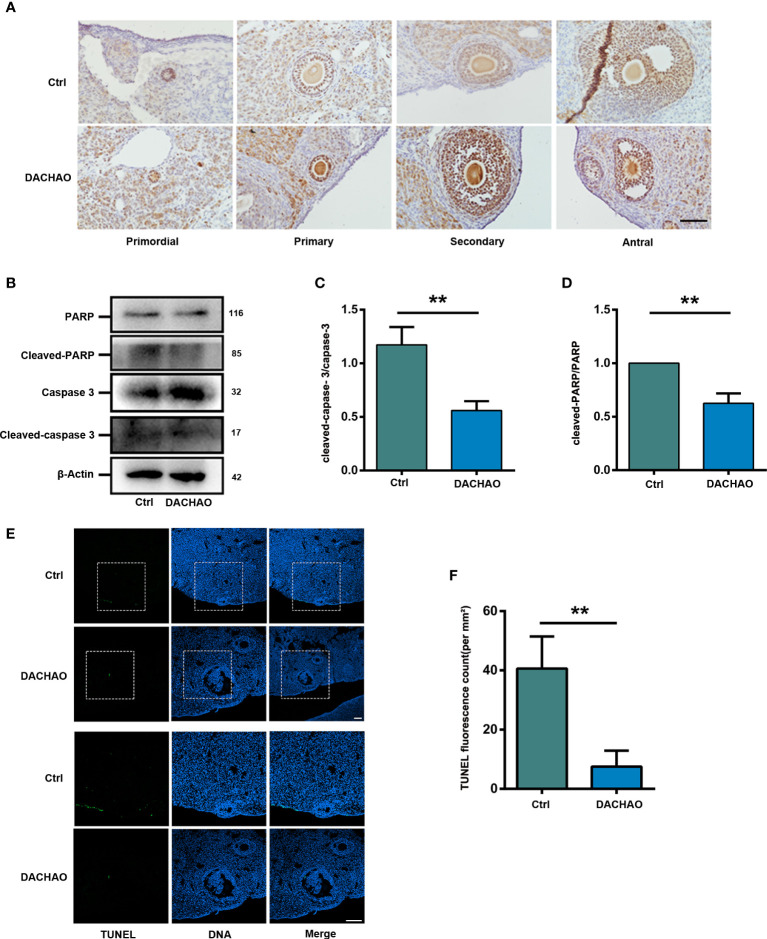
DACHAO improves fertility by promoting granulosa cell proliferation and reducing apoptosis in ovaries. **(A)** The morphology of PCNA immunohistochemical staining in different follicular development stages of mouse ovary in each group. **(B)** Western-blots of the expression of apoptosis-related proteins in mice ovaries in each group. **(C, D)** Gray value analysis of Western blots in **(B)** was quantified and is shown by cleaved-caspase-3-to-caspase-3 ratios, cleaved-PARP-to-PARP ratios (n=3/group). **(E)** TUNEL staining of ovaries of aged female mice in each group. **(F)** Statistical graph of the number of TUNEL staining positive signal points in the ovaries of aged female mice in each group (n=3/group). Data are presented as means ± SEM of three independent replicates. **P < 0.01, compared with the control group. n = the number of mice in each group. All bars = 50 μm.

Since follicular developed was improve in DACHAO treated mice, PCNA immunostaining was then performed to examine cell proliferation in the two groups. We found that in the treated group, the number of PCNA positive signal points around the follicles was more than that in the control group at growing follicle stage (**Figure 5A**). The results suggest that DACHAO administration can increase the proliferation of granulosa cells of growing follicles. To detect cell apoptosis in control and treated ovaries, western blot was performed to check the expression of apoptosis related proteins and the results showed that the expression of cleaved-caspase-3 and cleaved-PARP in the ovaries of DACHAO-treated mice was much lower than that of the control group (**Figures 5B–D**). Consistent with the western blot results, TUNEL staining on the ovaries from the two groups of mice further demonstrated the apoptosis in DACHAO treated group was lower than that in the control group (**Figures 5E, F**). Pictures from negative control and positive control (DNase-treated) for TUNEL assay are shown in **Figure S2**. This indicates that DACHAO administration can reduce the apoptotic levels in the aged ovaries. DACHAO enhanced the fertility by improving serum sex hormone levels and ovarian expression of hormone-related mRNAs.

**Figure 6:**
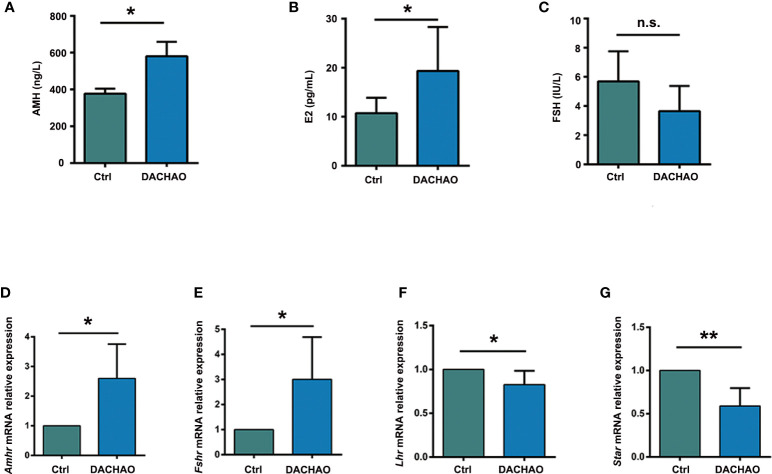
DACHAO enhanced the fertility by improving serum sex hormone levels and ovarian expression of hormone-related mRNAs. **(A–C)** Statistical analysis of serum AMH, E2, FSH values in aged female mice in each group (n=8/group). **(D–G)** Statistical analysis of relative expression of hormone-related mRNAs as Amhr, Fshr, Lhr and Star in ovaries of aged female mice in each group (n=3/group). Data are presented as means ± SEM of three independent replicates. *P < 0.05, **P < 0.01, and n.s. P≥0.05, compared with the control group. n = the number of mice in each group.

Since better follicular development was observed after DACHAO treatment, we then randomly selected mice in each group whose estrous cycle was at the interphase and collected venous blood samples for the measurement of serum AMH, FSH, and E2 levels by ELISA. The results showed that DACHAO treatment increased the serum AMH and E2 hormone levels in aged mice, with the value of AMH level increasing from 376.69 ng/L to 580.61 ng/L and the value of E2 level increasing from 10.73 pg/ml to 19.33 pg/ml. Although a decreased trend of FSH hormone level was observed in DACHAO treated mice, there is no-statistical significance between the two groups (**Figures 6A–C**). To test the biosafety of DACHAO, we additionally prepared ten 40W aged female ICR mice to gavage with 2 times the dose of DACHAO for one month. After the gavage, we collected venous blood samples of these mice and detected the liver and kidney function and blood lipid-related indicators between the two groups. The results showed that normal dose and high dose of DACHAO treatment for one month had no adverse effects on liver and kidney function and blood lipid levels in mice (**Figure S1B**). We then performed qRT-PCR analysis of steroid hormone-related mRNA expressions. DACHAO treatment demonstrated the increase of *Amhr* and *Fshr* mRNAs in the ovary (**Figures 6D–G**). Thus, the results suggest the improvement of granulosa cell function after DACHAO treatment.

### DACHAO improves the fertility by changing the expression of ovarian antioxidant and inflammation-related factors in the ovary

**Figure 7:**
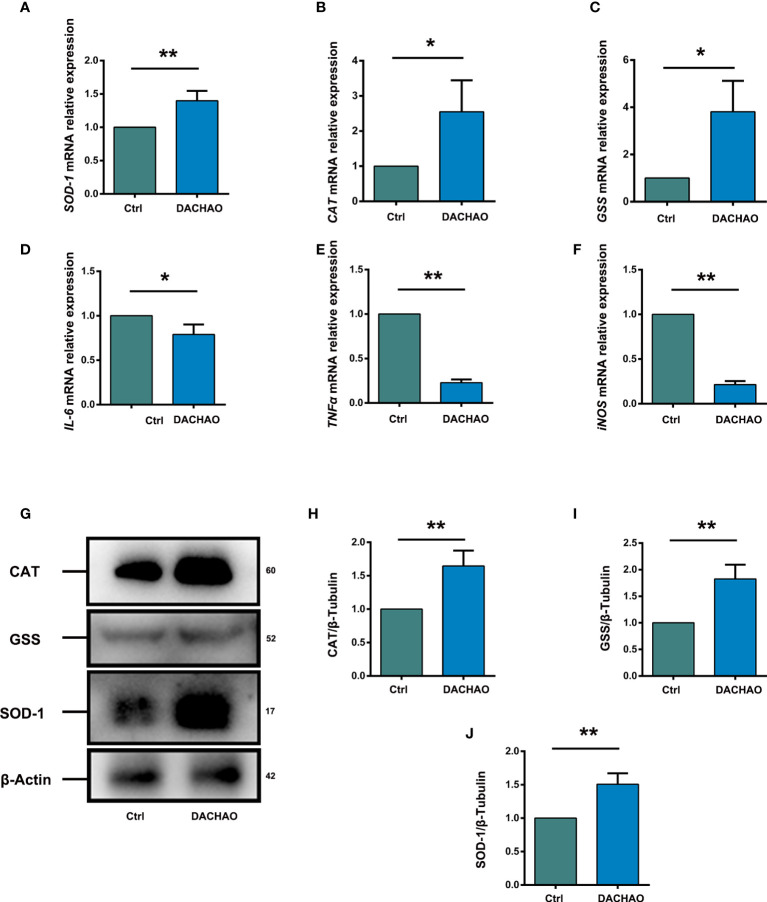
DACHAO improves the fertility changing the expression of ovarian antioxidant or inflammation-related factors. **(A–C)** Statistical analysis of relative expression of antioxidant factors SOD-1, CAT, GSS mRNA in ovaries of aged female mice in each group (n=3/group). **(D)** Western-blots of the expression of antioxidant factor-related proteins in mice ovaries in each group. **(E–G)** Gray value analysis of Western blots in **(D)** was quantified and is shown by SOD-1-to-β-Actin ratios, CAT-to-β-Actin ratios and GSS-to-β-Actin ratios (n=3/group). **(H–J)** Statistical analysis of relative expression of anti-inflammatory factors IL-6, TNFα, iNOS mRNA in ovaries of aged female mice in each group (n=3/group). Data are presented as means ± SEM of three independent replicates. *P < 0.05 and **P < 0.01, compared with the control group. n = the number of mice in each group.

Due to the improvement of mitochondria function in DACHAO treated oocytes, the effect of DACHAO on ovarian oxidative stress was evaluated by qRT-PCR and we found the increased expression of some antioxidant factors *SOD-1* (Superoxide dismutase 1), *CAT* (Catalase), and *GSS* (Glutathione synthetase) mRNAs in DACHAO treated ovaries (**Figures 7A–C**). Western blot further manifested the elevated protein levels of SOD-1, CAT, and GSS in the ovaries of DACHAO treated mice (**Figures 7D–G**). In addition, qRT-PCR also revealed the decreased expression of some inflammatory factors, such as *IL-6*, *TNFα*, and *iNOS* in DACHAO treated ovaries (**Figures 7H–J**). In conclusion, DACHAO treatment could alleviate the oxidative stress and the inflammatory environment in the aged ovary.

## Discussion

Many women are willing to postpone their marriage and/or childbirth due to social, economic, and cultural reasons. Age-related retardation of fertility have thus become serious challenges in the field of reproductive medicine, as aging may cause a decrease in both the quality and quantity of oocytes in women at advanced maternal age (36). High-dose follicle-stimulating hormone (FSH) regimens that applied to enhance the oocyte yield in IVF have largely been unsuccessful in older women due to poor ovarian response (POR) (37). Thus, new approaches or strategies to improve the fertility of aged women are urgently needed (38). Integrative medicine treatments using natural plant sources are considered to be effective for the improvement of the fertility in women at advanced maternal age and many herbal combinations have been reported to manage infertility symptoms in rodent models and humans (39–41). In this study, similar to the holistic treatment by traditional Chinese medicine prescriptions, we developed a strategy using a combination of several natural extracts from herbs. We then assessed the potential of this combination to improve the ovarian function in an aged female mouse model. To minimize any possible side effects, every single product we chose to utilize was natural food-grade plant extracts. Eugenol (the main component of clove), and quercetin (the main component of Sophora flower bud) are natural antioxidant, anti-inflammatory, and anti-aging agents, whose effects in age-related diseases have been extensively investigated. For instance, eugenol was reported to have a potential role in preventing and alleviating chronic diseases, such as cancer, inflammatory reactions, and other conditions (42). Eugenol has also been reported to attenuate brain D-galactose-induced aging-related oxidative alterations in rats (43). moreover, quercetin was found to improve cognitive disorder in aging mice by inhibiting NLRP3 inflammasome activation (44).

The therapeutic impact of these natural medicines on ovarian-related diseases has also been assessed. Eugenol was shown to improve tissue damage and oxidative stress after ovarian torsion in adult female rats (45). A previous study showed that clove extract promoted blastocyst development in frozen mouse oocytes of *in vitro* fertilized embryos (24). In addition, in several *in vitro* studies on animal and human granulosa cells, quercetin treatment reduced the percentage of early apoptotic cells, enhanced the quality of oocytes, and improved the subsequent embryo development (26, 46, 47). A novel protein, DOI, isolated from Chinese yam was reported to stimulate estradiol biosynthesis in rat ovarian granulosa cells, with potential to treat menopausal syndrome (48).

DACHAO is a healthy product that contains all aforementioned components of food grade and natural plant origin. In our study, we found that DACHAO improved the fertility in aged mice *via* several mechanisms. Our results showed that the administration of DACHAO can increase the number of ovulated oocytes in aged female mice and moreover improve oocyte quality, compared with the control group. In other words, DACHAO improves oocyte quality in aged female mice both qualitatively and quantitatively. As expected, we found that administration of DACHAO promoted the fertility in aged female mice, by both *in vitro* fertilization and by natural mating. In addition, our data showed that the DACHAO administration in aged female mice improved the quality of embryos and increased the growth rate of their offspring. To investigate the mechanisms by which DACHAO improves the fertility of aged mice, we conducted further research and found that DACHAO administration promoted the proliferation of the granulosa cells around the follicles and reduced the level of apoptosis in the ovaries. Further data revealed that DACHAO administration improved the hormone level and increased the expressions antioxidant factors in aged female mice. The mRNA expression levels of ovarian inflammation-related factors in the mice in the DACHAO administration group were lower than those in the control group. In conclusion, DACHAO treatment improves the hormone level and reduces the oxidative stress and inflammatory levels in the ovary of aged mice, providing a more favorable microenvironment for oocyte growth and maturation. This is probably related to the estrogen-like effects of yam and the antioxidant and anti-inflammatory effects of clove and quercetin which are contained in DACHAO. Our study provides new evidence for the development of a food grade health product for fertility improvement in aging mice with a high potential and a harmless protocol of administration. Nevertheless, this product and protocol have not yet been applied in women at advanced maternal age. Therefore, future cohort study is required to evaluate the effect of DACHAO on the reduction of fertility in women at middle-age. We anticipate more strategies to appear in the form of “food grade health products” that can help women at advanced maternal age who are willing to get pregnant and give safe birth. We consider that this study also provides insights and ideas for therapies of premature ovarian insufficiency.

## Conclusions

In general, we have developed a food grade health product that can effectively improve the fertility of aged female mice, mainly *via* improving serum estrogen levels, promoting follicle growth and maturation, increasing the expression of antioxidant factors, decreasing the expression of inflammation-related factors, and reducing the level of apoptosis in the ovaries associated with age. Next, we intend to conduct a cohort study to evaluate the effect of DACHAO on the reduced fertility in women at middle-age.

## Data availability statement

The raw data supporting the conclusions of this article will be made available by the authors, without undue reservation.

## Ethics statement

The animal study was reviewed and approved by the Committee on the Ethics of Nanjing Medical University.

## Author contributions

JL and TW conceptualized the study. WL and JL led the experimental design and wrote the manuscript. WL, XW, YC, HZ and JZ performed the experiments. JC and TW corrected the manuscript. All authors contributed to the article and approved the submitted version.

## Funding

This work was supported by the National Key R&D Program of China (2018YFC0310600).

## Conflict of interest

TW was employed by Jiangsu Simcere Pharmaceutical Co., Ltd. JC was employed by Hainan Leyun Biotechnology Co., Ltd.

The remaining authors declare that the research was conducted in the absence of any commercial or financial relationships that could be construed as a potential conflict of interest.

## Publisher’s note

All claims expressed in this article are solely those of the authors and do not necessarily represent those of their affiliated organizations, or those of the publisher, the editors and the reviewers. Any product that may be evaluated in this article, or claim that may be made by its manufacturer, is not guaranteed or endorsed by the publisher.

## Supplementary material

Supplementary Figure 1 One month after DACHAO treatment, there was no obvious side effect on the body weight and blood biochemical indices of mice. **(A)** The body weights of mice from different groups after gavage. (n=8 or 20/group). **(B)** The blood biochemical indices of mice from different groups after gavage. (n=5/group). *P < 0.05, **P < 0.01, ***P < 0.001, and n.s. P≥0.05, compared with the control group. n = the number of mice in each group.

Supplementary Figure 2 Pictures from negative control and positive control (DNase-treated) for TUNEL assay of ovarian sections.

Supplementary Table 1 All of the primer sequences used in the study are listed here.
