# Supplementary figures and images for: A combination containing natural extracts of clove, Sophora flower bud, and yam improves fertility in aged female mice via multiple mechanisms
Source: Front Endocrinol (Lausanne). 2022 Nov 22;13:945690. doi: 10.3389/fendo.2022.945690 (PMC9724743; doi:10.3389/fendo.2022.945690)

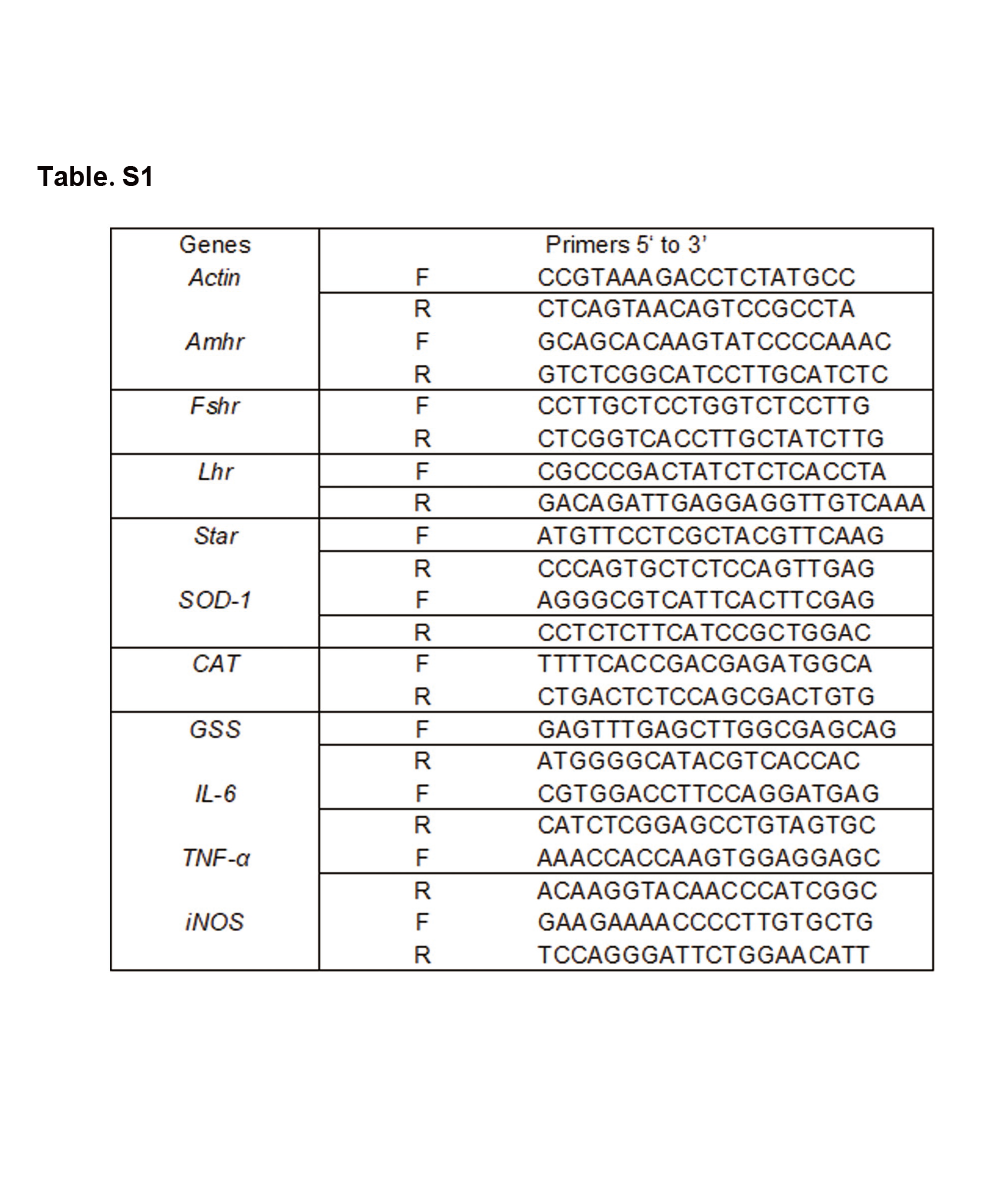

Supplement: Supplementary Figure 1 — One month after DACHAO treatment, there was no obvious side effect on the body weight and blood biochemical indices of mice. (A) The body weights of mice from different groups after gavage. (n=8 or 20/group). (B) The blood biochemical indices of mice from different groups after gavage. (n=5/group). *P < 0.05, **P < 0.01, ***P < 0.001, and n.s. P≥0.05, compared with the control group. n = the number of mice in each group. [file Image_1.tif]

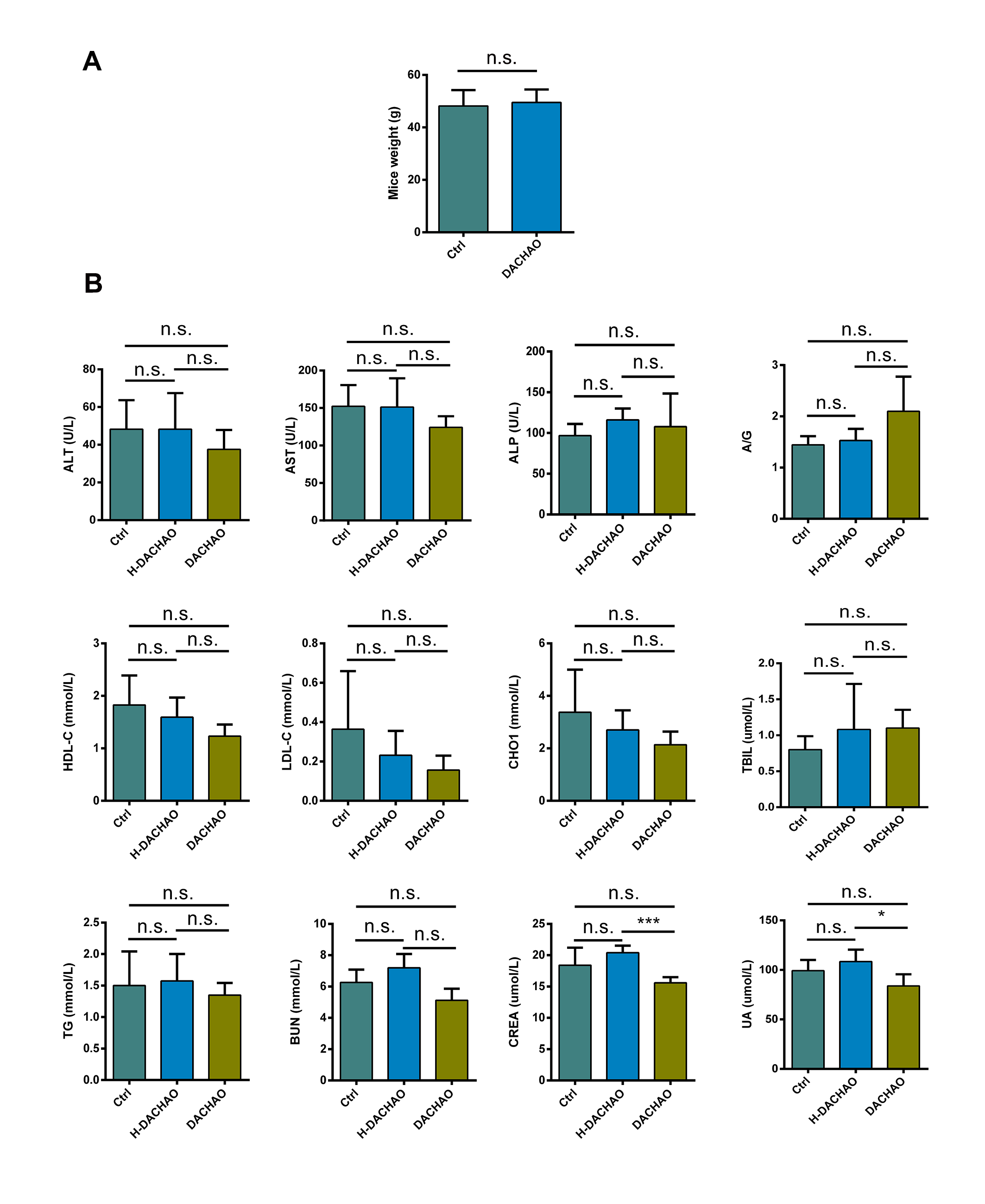

Supplement: Supplementary Figure 2 — Pictures from negative control and positive control (DNase-treated) for TUNEL assay of ovarian sections. [file Image_2.tif]

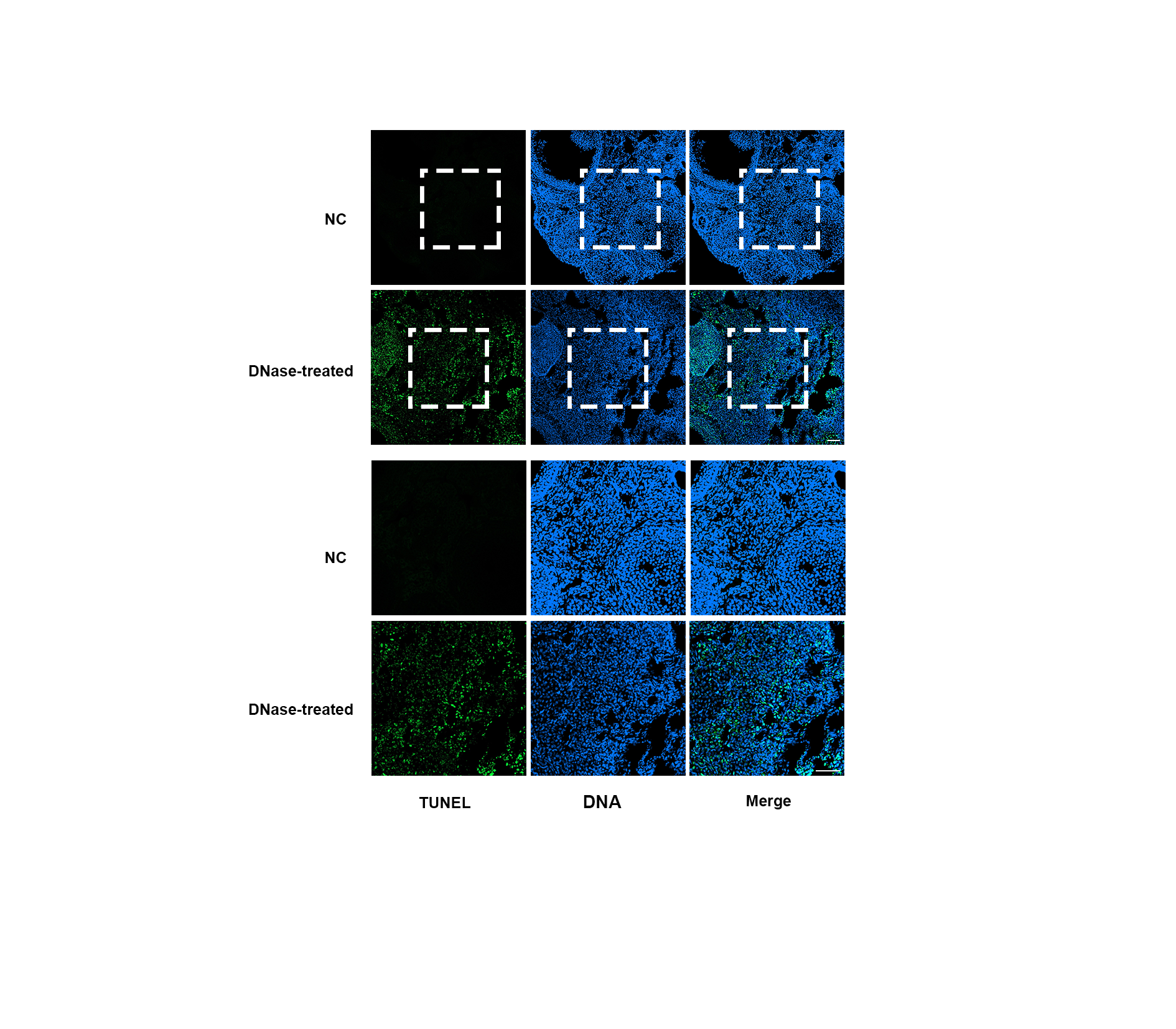

Supplement: Supplementary Table 1 — All of the primer sequences used in the study are listed here. [file Image_3.tif]
